# Efficient Identification of the Forest Tree Species in Aceraceae Using DNA Barcodes

**DOI:** 10.3389/fpls.2016.01707

**Published:** 2016-11-16

**Authors:** Yu-Wei Han, Dong Duan, Xiong-Feng Ma, Yun Jia, Zhan-Lin Liu, Gui-Fang Zhao, Zhong-Hu Li

**Affiliations:** ^1^Key Laboratory of Resource Biology and Biotechnology in Western China, Ministry of Education, College of Life Sciences, Northwest UniversityXi'an, China; ^2^State Key Laboratory of Cotton Biology, Institute of Cotton Research, Chinese Academy of Agricultural SciencesAnyang, China

**Keywords:** Aceraceae, chloroplast DNA, barcoding markers, species identification, *ITS*

## Abstract

Aceraceae is a large forest tree family that comprises many economically and ecologically important species. However, because interspecific and/or intraspecific morphological variations result from frequent interspecific hybridization and introgression, it is challenging for non-taxonomists to accurately recognize and identify the tree species in Aceraceae based on a traditional approach. DNA barcoding is a powerful tool that has been proposed to accurately distinguish between species. In this study, we assessed the effectiveness of three core standard markers (*mat*K, *rbc*L and *ITS*) plus the chloroplast locus *trn*S*-trn*G as Aceraceae barcodes. A total of 231 sequences representing 85 species in this forest family were collected. Of these four barcode markers, the discrimination power was highest for the *ITS* (I) region (50%) and was progressively reduced in the other three chloroplast barcodes *mat*K (M), *trn*S-*trn*G (T) and *rbc*L (R); the discrimination efficiency of the *ITS* marker was also greater than any two-locus combination of chloroplast barcodes. However, the combinations of *ITS* plus single or combined chloroplast barcodes could improve species resolution significantly; T+I (90.5% resolution) and R+M+T+I (90.5% resolution) differentiated the highest portion of species in Aceraceae. Our current results show that the nuclear *ITS* fragment represents a more promising DNA barcode marker than the maternally inherited chloroplast barcodes. The most efficient and economical method to identify tree species in Aceraceae among single or combined DNA barcodes is the combination of T+I (90.5% resolution).

## Introduction

The forest plant family Aceraceae is the largest family of broad-leaved deciduous trees in the Northern Hemisphere (Wolfe and Tanai, 1987). This forest family includes two genera, *Acer* L. and *Dipteronia* Oliv., with approximately 140 species. Maple species are widespread and play key roles in local forest ecosystems. In addition, because of their rapid growth rates, great value for landscaping and adaptability to various harsh environmental conditions, maples are widely cultivated and exploited (Saeki and Murakami, 2009). Maples have become a model organism due to their fascinating sex systems (e.g., duodichogamy, protandry, and protogyny) and ease of use in biological research (Shang et al., 2012). Simultaneously, wild maple species provide an important breeding resource for the future from an economic view point. However, the recognition and identification of wild maple species based on traditional morphological approaches is difficult due to their extensive interspecific and/or intraspecific hybridization and introgression (Li et al., 2006; Li, 2011), which causes transitional intraspecific morphological characteristics in Aceraceae. Nevertheless, the recently proposed DNA barcoding method is one possible way to resolve this problem (Hebert et al., 2003).

DNA barcoding aims to achieve rapid and accurate species recognition by sequencing a short DNA sequence or a few DNA regions (Hebert et al., 2003; Hebert and Gregory, 2005; Kress et al., 2005; Hollingsworth et al., 2009; Li et al., 2011). This technology was first developed to diagnose animal species, and “the mitochondrial gene cytochrome *c* oxidase I (*COI*) can be served as the core of a global bio-identification system for animals” (Hebert et al., 2003). Increasingly, studies have demonstrated that the *COI* gene fragment is highly efficient for discrimination among animal species, including amphibians (Vences et al., 2005), birds (Hebert et al., 2004), fish (Ward et al., 2009), and insects (Pons et al., 2006). In plants, however, frequent recombination and low mutation rates are the main restrictions in finding efficient mitochondrial barcode markers (Cho et al., 1998, 2004; Hollingsworth et al., 2011), and the search for suitable candidates has focused on chloroplast and nuclear DNA markers (Kress et al., 2005; CBOL Plant Working Group, 2009; Li et al., 2011; Yan et al., 2015), although it is not easy to amplify and sequence universal nuclear gene primers across different angiosperm taxa. Numerous studies have suggested that four standard barcodes, *mat*K, *rbc*L, *trn*H-*psb*A, and *ITS*, should be used as core barcode markers for the molecular identification of land plants (Hollingsworth et al., 2011; Li et al., 2011). In addition to choosing DNA barcoding fragments, collecting many individuals from different populations within a species is also important for establishing a reference database for universal application (Bolson et al., 2015; Guo et al., 2016).

**Figure 1:**
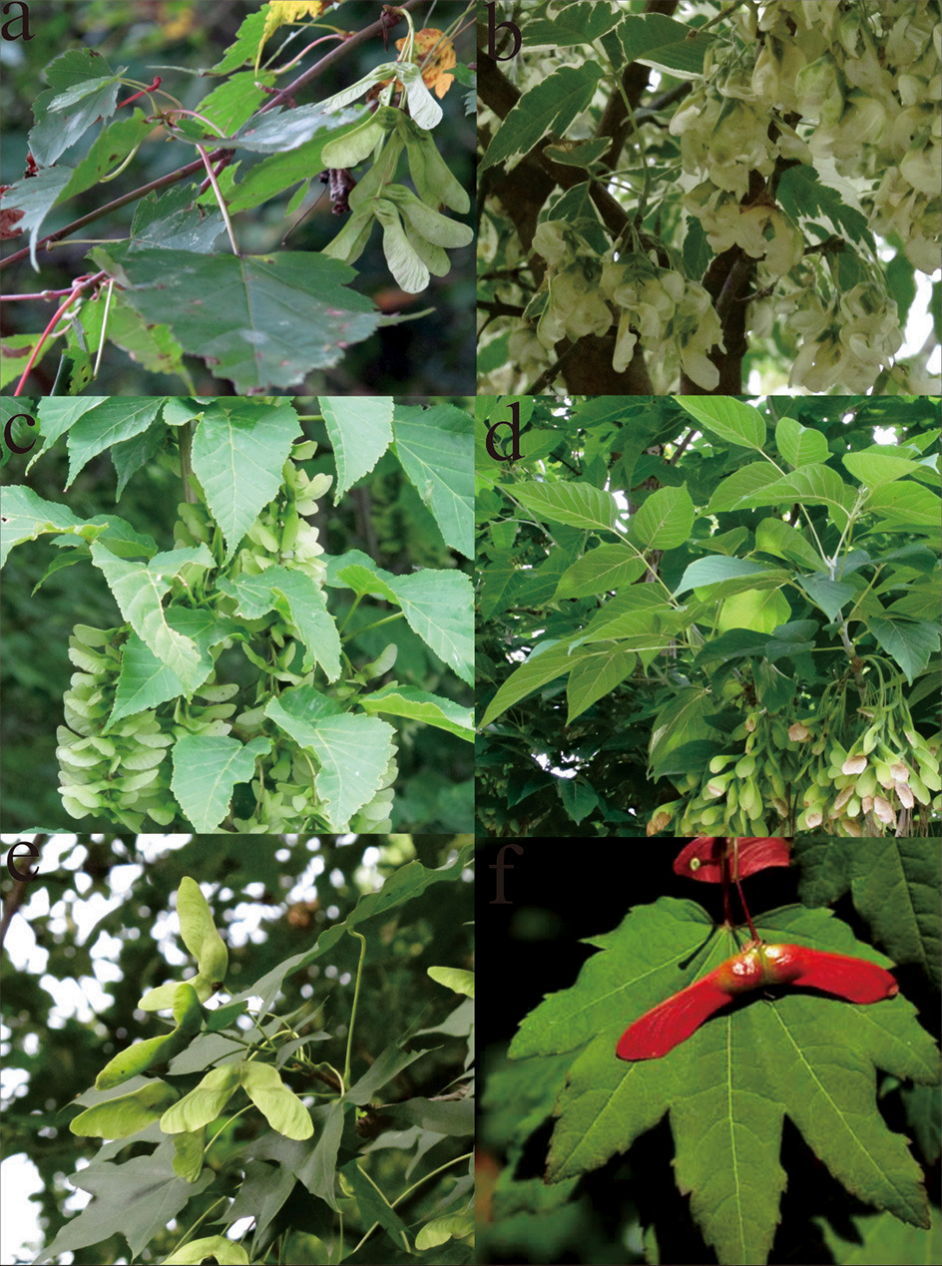
**The variations and similarities in the morphology of maple species. a,** *Acer ginnala*; **b**, *A. negundo* var. *variegatum*; **c**, *A. davidii*; **d**, *A. negundo*; **e**, *A. truncatum*; **f**, *A. circinatum*: from http://oregonstate.edu/trees/broadleaf_genera/species/maple_spp.html.

Maples are important ornamental and horticultural woody species in the Northern Hemisphere. They are widely distributed in the temperate and subtropical regions of northern Africa, eastern Asia, Europe, and eastern North America (van Gelderen et al., 1994). China is a modern center of geographic distribution and diversification because approximately 70% of the species in Aceraceae occur in this country. According to leaf shapes, inflorescence types and elongated winged fruits (Figure 1), different classification systems for Aceraceae have been proposed. Pax divided the genus *Acer* into 13 sections in his classification system of *Acer* (Pax, 1902). In Murray (1970), the genus *Acer* was classified into 24 sections. More recently, Xu (1996) recognized 23 sections and discussed the phylogenetic relationships among species of Aceraceae.

In recent years, the identification of closely related maple species has been a process of continuous development. Previous studies have demonstrated that restriction fragment length polymorphism (RFLP) and amplified fragment-length polymorphism (AFLP) markers are useful for differentiation of the sections *Arguta, Cissifolia, Lithocarpa*, and *Spicata* in Aceraceae (Hasebe et al., 1998). A phylogenetic study has shown that two chloroplast DNA loci, *psb*M-*trn*D and *trn*D-*trn*T, could be used to discriminate between the genera *Acer* and *Dipteronia* (Li et al., 2006), and studies based on the combined datasets of the nuclear *ITS* and the chloroplast noncoding *trn*L-*trn*F region (Tian et al., 2002) suggest that the genus *Dipteronia* might be paraphyletic with *D. deyerana* embedded in *Acer*. Furthermore, a recent phylogenetic reconstruction suggested that the *ITS* sequence in Aceraceae evolved rapidly, and this might lead to high species resolution in this forest family (Grimm et al., 2006).

However, many maple species have complex sex systems, and since seeds and pollen are dispersed by wind and birds, interspecific hybridization and introgression occur extensively between closely related species, causing transitional intraspecific morphological variations, such as similar shapes of leaves, inflorescences and samaras (Guo et al., 2014; Liu C. et al., 2014). Therefore, it is difficult to recognize and delimitate maple species accurately by available morphological characteristics (Tian et al., 2002). In the current study, we used four DNA candidate barcodes that had been proposed previously, three chloroplast markers (*rbc*L, *mat*K, and *trn*S-*trn*G), and one nuclear *ITS* fragment, to differentiate 231 samples representing 85 Aceraceae species. Our primary objectives were to: (1) test the universality of the four DNA markers in Aceraceae; (2) examine the resolution of these four markers alone or in combination and evaluate the most appropriate marker among these barcodes to distinguish between species within the family Aceraceae; and (3) establish a reference database to facilitate future identification of forest trees.

## Materials and methods

### Plant materials

A total of 231 sequences representing 85 species in Aceraceae were collected and analyzed. Hybridization and/or introgression are very common in Aceraceae, which may have affected the performance of the tested DNA barcodes. In the current study, we did not sample the complicated introgression species in our analysis of DNA barcoding. Xu (1996) included 22 sections in the genus *Acer*: *Acer, Distyla, Ginnala, Glabra, Goniocarpa, Hyptiocarpa, Integrifolia, Macrantha, Macrophylla, Microcarpa, Palmata, Parviflora, Pentanphylla, Platanoidea, Trifoliata, Rubra, Saccarodendron, Lithocarpa, Carpinifolia, Arguta, Cissifolia*, and *Negundo*. We sampled the leaves of 85 trees representing 21 maple species from Shaanxi, Gansu, Taiwan, and Yunnan, in China (Table 1). In total, 2–5 individuals per species were sampled from 1 to 4 populations in the field. Fresh leaves were dried and stored in silica gel, and the longitude, latitude and altitude of each collection site (population) were recorded using a GIS unit (Garmin, Taiwan). The voucher specimens were stored in the Key Laboratory of Resource Biology and Biotechnology in Western China (KLRBBWC), Northwest University. To thoroughly assess the efficiency of species identification by single or combined DNA barcodes, we also downloaded 108, 81 and 146 sequences of *rbc*L, *mat*K and *ITS* fragments that represented 45, 33, and 47 Aceraceae species, respectively, from GenBank (Table S1).

**Table 1:** **Locations and descriptions of Aceraceae species**.

**Section**	**Species**	**Origin**	**Sampling size**	**Voucher ID**	**Longitude**	**Latitude**	**Alt. (m)**
Section *Palmata* Pax	*Acer palmatum* Thunb.	Xi'an (XA), Shaanxi	2	LIZH-2015023	E108°57′25.2″	N34°12′30.5″	419
	*A. Palmatum* Thunb. var. *palmatum*	Xi'an (XA), Shaanxi	5	LIZH-2015001	E108°54′14.5″	N34°14′53.1″	414
	*A. robustum* Pax.	Xi'an (XA), Shaanxi	5	LIZH-2015052	E108°57′25.2″	N34°12′30.5″	419
Section *Platanoidea* Pax	*A. mono* Maxim.	Baoji (BJ), Shaanxi	2	LIZH-2014036	E106°40′27.8″	N35°02′19.0″	1669
		Baoji (BJ), Shaanxi	1	LIZH-2014058	E107°45′27.2″	N34°09′32.6″	718
	*A. miaotaiense* Tsoong.	Xi'an (XA), Shaanxi	4	LIZH-2014059	E108°57′25.2″	N34°12′30.5″	419
	*A. truncatum* Bunge.	Xi'an (XA), Shaanxi	5	LIZH-2015024	E108°57′25.2″	N34°12′30.5″	419
Section *Negundo* (Boehmer) Maxim	*A. negundo* Linn.	Xi'an (XA), Shaanxi	4	LIZH-2014196	E108°57′25.2″	N34°12′30.5″	419
Section *Arguta* (Rehd.) E. Murray	*A. tetramerum* Pax var. *betulifolium* (Maxim.) Rehd.	Lanzhou (LZ), Gansu	5	LIZH-2013022	E104°03′25.8″	N35°46′35.2″	2592
Section *Cissifolia* Koidzumi	*henryi* Pax.	Xi'an (XA), Shaanxi	5	LIZH-2013002	E108°57′25.2″	N34°12′30.5″	419
Section *Ginnala* Nakai	*A. ginnala* Maxim. subsp. *theiferum* (Fang) Fang.	Xi'an (XA), Shaanxi	5	LIZH-2014035	E108°57′25.2″	N34°12′30.5″	419
	*A. ginnala* Maxim.	Chongxin (CX), Gansu	2	LIZH-2014068	E106°58′30.4″	N35°09′34.3″	1221
Section *Trifoliata* Pax	*A. mandshuricum* Maxim. subsp. *kansuense* (Fang et C. Y. Chang) Fang.	Ankang (AK), Shaanxi	3	LIZH-2014045	E108°50′0.8″	N33°46′47.8″	1948
	*A. griseum* (Franch.) Pax.	Baoji (BJ), Shaanxi	5	LIZH-2015091	E106°40′27.8″	N35°02′19.0″	1668
Section *Macrantha* Pax	*A. morrisonense* Hayata.	Hualian (HL), Taiwan	5	LIZH-2014132	E121°16′53.0″	N24°10′53.0″	3404
	*A. davidii* Franch.	Hanzhong (HZ), Shaanxi	1	LIZH-2015042	E107°52′8.3″	N32°28′8.8″	1224
		Baoji (BJ), Shaanxi	1	LIZH-2015030	E107°44′57.8″	N34°26′13.7″	677
		Baoji (BJ), Shaanxi	1	LIZH-2014114	E106°40′27.8″	N35°02′19.0″	1668
		Shangluo (SL), Shaanxi	1	LIZH-2015005	E109°09′2.8″	N33°50′8.3″	1738
	*A. kawakamii* Koidzumi.	Hualian (HL), Taiwan	5	LIZH-2015054	E121°16′53.0″	N24°10′53.0″	3405
Section *Integrifolia* Pax	*A. buergerianum* Miq.	Shangluo (SL), Shaanxi	5	LIZH-2015153	E109°09′2.8″	N33°50′8.3″	1738
	*A. oblongum* Wall. ex DC.	Shangluo (SL), Shaanxi	5	LIZH-2014033	E109°09′2.8″	N33°50′8.3″	1738
Section *Microcarpa* Pojark.	*A. oliverianum* Pax	Xi'an (XA), Shaanxi	2	LIZH-2015235	E108°31′36.3″	N33°55′29.3″	1337
	*Dipteronia dyeriana* Henry.	Binbian (BB), Yunnan	1	LIZH-ZT-201505	E103°47′31.3″	N23°15′16.3″	2001
		Mengzi (MZ),Yunnan	1	LIZH-ZT-201522	E103°46′53.0″	N23°24′4.0″	2117
		Wenshan (WS),Yunnan	1	LIZH-ZT-201528	E103°57′16.0″	N23°16′27.0″	2011
		Wenshan (WS),Yunnan	1	LIZH-ZT-201532	E103°58′29.1″	N23°20′52.8″	2204
	*D. sinensis* Oliv.	Xi'an (XA), Shaanxi	2	LIZH-ZT-201501	E108°57′25.2″	N34°12′30.5″	419

### DNA extraction, PCR amplification, and sequencing

Total genomic DNA of the collected samples was extracted from approximately 20 mg of silica-dried leaves using a modified cetyltrimethylammonium bromide (CTAB) method (Doyle, 1987). Amplification of DNA regions was performed by standard polymerase chain reaction (PCR). The primer sequences and thermocycling conditions of PCR amplification are listed in Table 2. The PCR reactions were conducted in a 25 μL mixture system containing 12.5 μL 2 × Taq PCR mix (Runde, Xi'an, China), 1.0 μL of each primer (5 μmol/L), 10.5 μL ddH_2_O, and 1.0 μL template DNA (30–50 ng).

**Table 2:** **List of primers and reaction conditions for candidate barcodes**.

**DNA region**	**Primer pairs**	**Primer sequences (5′-3′)**	**Thermocycling conditions**
*rbc*L	*rbc*l1F	ATGTCACCACAAACAGAAAC	94°C 5 min, 36 cycles (94°C 40 s, 52°C 40 s, 72°C 1 min), 72°C 10 min
	*rbc*l724R	TCGCATGTACCTGCAGTAGC	
*mat*K	*mat*K3Fkim	CGTACAGTACTTTTGTGTTTACGAG	94°C 5 min, 36 cycles (94°C 40 s, 52°C 40 s, 72°C 1 min), 72°C 10 min
	*mat*K1Rkim	ACCCAGTCCATCTGGAAATCTTGGTTC	
*trn*S-*trn*G	*trn*S-*trn*GF	GCCGCTTTAGTCCACTCAGC	94°C 5 min, 36 cycles (94°C 40 s, 56°C 40 s, 72°C 1 min), 72°C 10 min
	*trn*S-*trn*GR	GAACGAATCACACTTTTACCAC	
*ITS*	ITS4	TCCTCCGCTTATTGATATG	94°C 5 min, 36 cycles (94°C 40 s, 52°C 40 s, 72°C 1 min), 72°C 10 min
	ITS5	GGAAGGAGAAGTCGTAACAAGG	

### Data analysis

All sequence assemblies and adjustment were performed using MEGA software version 6.0 (Tamura et al., 2013). For the alignments of each gene and the combinations of multiple loci, the parameters were: (1) multiple alignment, gap opening penalty 15, gap extension penalty 6.66; 2) DNA weight matrix IUB; (3) transition weight 0.5; and (4) delay divergent cutoff 30%. In total, for the *rbc*L (R) region, 193 sequences represented 66 species; for *mat*K (M), 166 sequences represented 54 species; for *trn*S-*trn*G (T), 85 sequences represented 21 species; for *ITS* (I), 231 sequences represented 68 species; for the R+M regions, 134 sequences represented 47 species; for the M+T, R+T, T+I, R+M+T, and R+M+T+I regions, 85 sequences represented 21 species; for the R+I regions, 121 sequences represented 46 species; and for the M+I regions, 119 sequences represented 41 species (Table S1; Supplementary Material Data Sheet). In particular, insertions/deletions (indels) and single nucleotide polymorphisms (SNPs) were calculated using DnaSP version 5.10.01 (Librado and Rozas, 2009). To evaluate species discrimination success, we applied two different methods of analysis for single markers and all possible combinations of markers.

### PWG-distance method

The barcoding gap was a significant factor used to test for appropriate barcode markers, which show high interspecific, but low intraspecific genetic divergence. The CBOL Plant Working Group (2009) recommended the PWG-Distance method to calculate distances from pairwise alignments by counting unambiguous base substitutions, and pairwise nucleotide genetic distances were based on the Kimura 2-parameter (K2P) nucleotide evolutionary model obtained from the MEGA 6.0 program (Tamura et al., 2013). We suggest that successful species discrimination was confirmed if the minimum uncorrected interspecific p-distance involving a species was larger than its maximum intraspecific distance (CBOL Plant Working Group, 2009).

### Tree-building method

Tree-Building is a method that uses the program MEGA 6.0 (Tamura et al., 2013) to construct a Neighbor-Joining (NJ) tree for each single marker and the different combinations of barcode markers under the Kimura 2-parameter substitution model (Hall, 2013). The bootstrap support of the NJ tree was assessed using 1000 replicates. The sampled species was considered successfully discriminated if all individuals of a species formed a monophyletic group in the phylogenetic tree (Hollingsworth et al., 2009). The ratio of successfully identified species to all sampled species was calculated as the proportion of species that were discriminated.

## Results

### Sequence characterization

PCR amplification succeeded for all DNA samples for the four DNA barcoding regions, *ITS, mat*K, *rbc*L, and *trn*S-*trn*G. However, there were two difficulties in sequencing and alignment. First, problems were encountered in the sequencing of the *trn*S-*trn*G region, where a mono-nucleotide repeat (poly-A) fragment in the middle of this marker led to sequencing failure for the latter half of this region in most of the individuals. Therefore, this relatively short chloroplast region was sequenced with both forward and reverse primers and then assembled. Second, several sequences of *ITS* were extremely hard to align due to its considerable number of variable sites, which required us to carefully check and manually edit the sequences. Eventually, the four regions were successfully amplified and sequenced in 21 sampled species. Eighty-five new Aceraceae sequences were obtained (KX264925-KX264991), and we also downloaded 45 other sequences of Aceraceae species from GenBank (Table S1). In total, 231 sequences of 85 species in Aceraceae were analyzed. The aligned lengths of the four DNA barcodes ranged from 346 bp (*ITS*) to 636 bp (*mat*K), and mutation sites (SNPs) were lowest for *rbc*L (27) and highest for *ITS* (137). Compared to the other markers, *rbc*L was the most highly conserved, with the fewest mutation sites and indels (0). A comparison of four single barcoding regions and one combination of all chloroplast regions is shown in Table 3.

**Table 3:** **Evaluation of four DNA markers and the combination of the three chloroplast DNA regions**.

**Region**	**Aligned seq. length (bp)**	**No. SNPs**	**% SNP**	**No. inDels**	**Interspecific distance (mean)**	**Intraspecific distance (mean)**	**Rate (%) PWG**	**Rate (%) NJ**
*ITS*	346	137	39.6	21	0.0474	0.0062	61.7	61.7
*rbc*L	497	27	5.43	0	0.0044	0.0004	10.26	10.26
*mat*K	636	69	10.85	1	0.0086	0.0008	42.42	42.42
*trn*S-*trn*G	366	40	10.93	44	0.0268	0	33.3	33.3
Three cp region	1499	93	6.2	45	0.0111	0.00002	71.4	71.4

*Aligned seq. length, length of PCR product amplified with the given primers in bp; cp, chloroplast; No. SNPs, the number of SNPs; %SNP, percentage SNP calculated as the number of SNPs in relation to the longest sequence length; No. inDels, the number of Insertions/Deletions; Interspecific distance (mean), the barcoding gap between species; Intraspecific distance (mean), the barcoding gap within species; Rate (%), percentage successful discrimination species calculated as the number of success discrimination species in relation to the total species; PWG, PWG-Distance method; NJ, Tree-Building method (Neighbor-Joining tree)*.

### Interspecific and intraspecific variability

**Figure 2:**
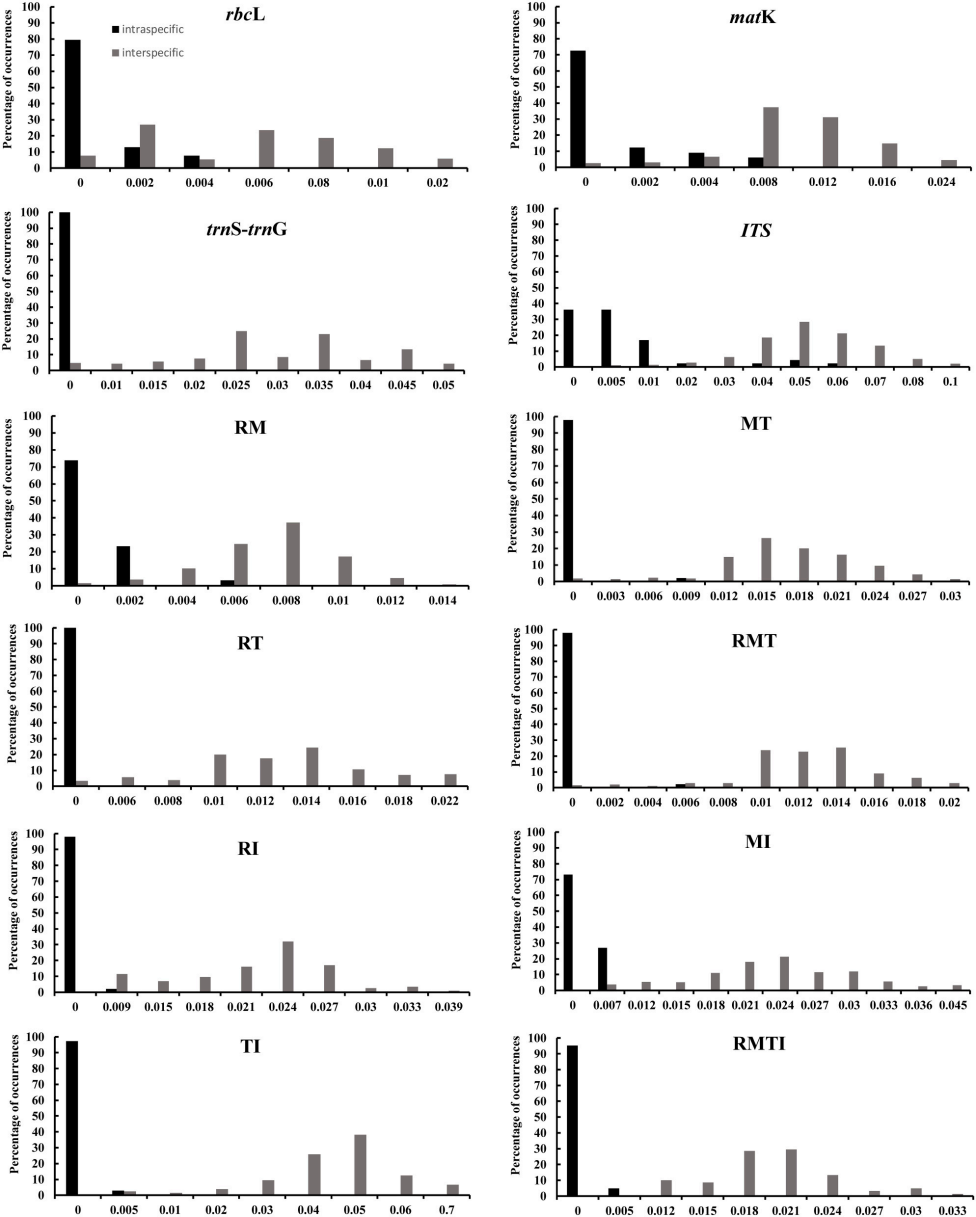
**Histograms of the frequencies (y-axes) of pairwise intraspecific (black bars) and interspecific (gray bars) divergences based on the K2P distance (x-axes) for individual and combined ***rbc***L (R), ***mat***K (M), ***trn***S-***trn***G (T), and ***ITS*** (I) markers**.

We investigated variability of the four DNA markers for all Aceraceae species, and all DNA regions showed higher genetic variability between than within species (Table 3). The nuclear *ITS* region showed the highest interspecific sequence divergence (4.74%), followed by *trn*S-*trn*G (2.68%), *mat*K (0.86%) and *rbc*L (0.44%). In addition, the intraspecific sequence divergence was also higher for *ITS* (0.62%) and lower for *trn*S-*trn*G (0%) between all the detected single-locus barcodes. The barcoding gap between interspecific and intraspecific distances was graphed based on the K2P model for each candidate barcode marker and the combinations of markers (Figure 2). Our results demonstrated that the combination of all four markers (*rbc*L+*mat*K+*trn*S-*trn*G+*ITS*, RMTI) showed the highest interspecific gap (divergence), and light overlaps without distinct gaps were found for single markers and/or combinations of the candidate loci (Figure 2).

### Species discrimination

**Figure 3:**
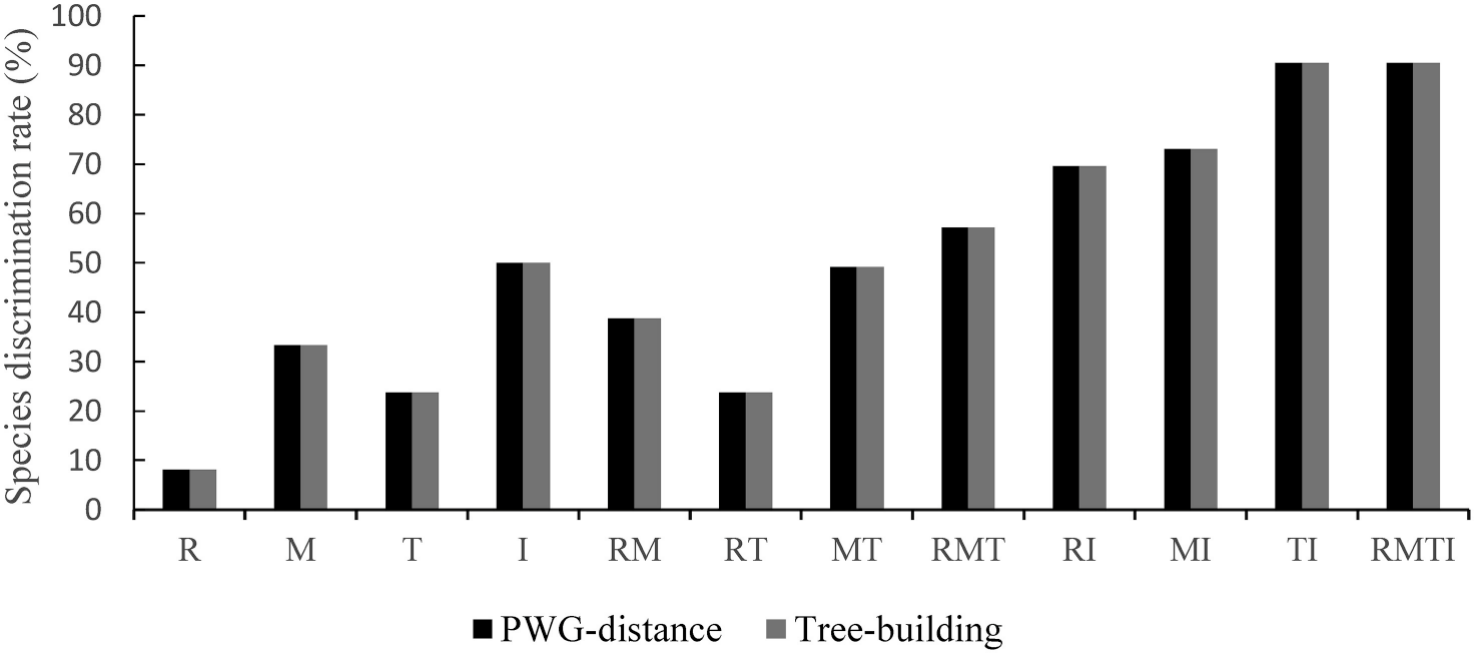
**Species discrimination rate of all tested single- and multi-locus barcodes in Aceraceae**. R, *rbc*L; M, *mat*K; T, *trn*S-*trn*G; I, *ITS*.

**Figure 4:**
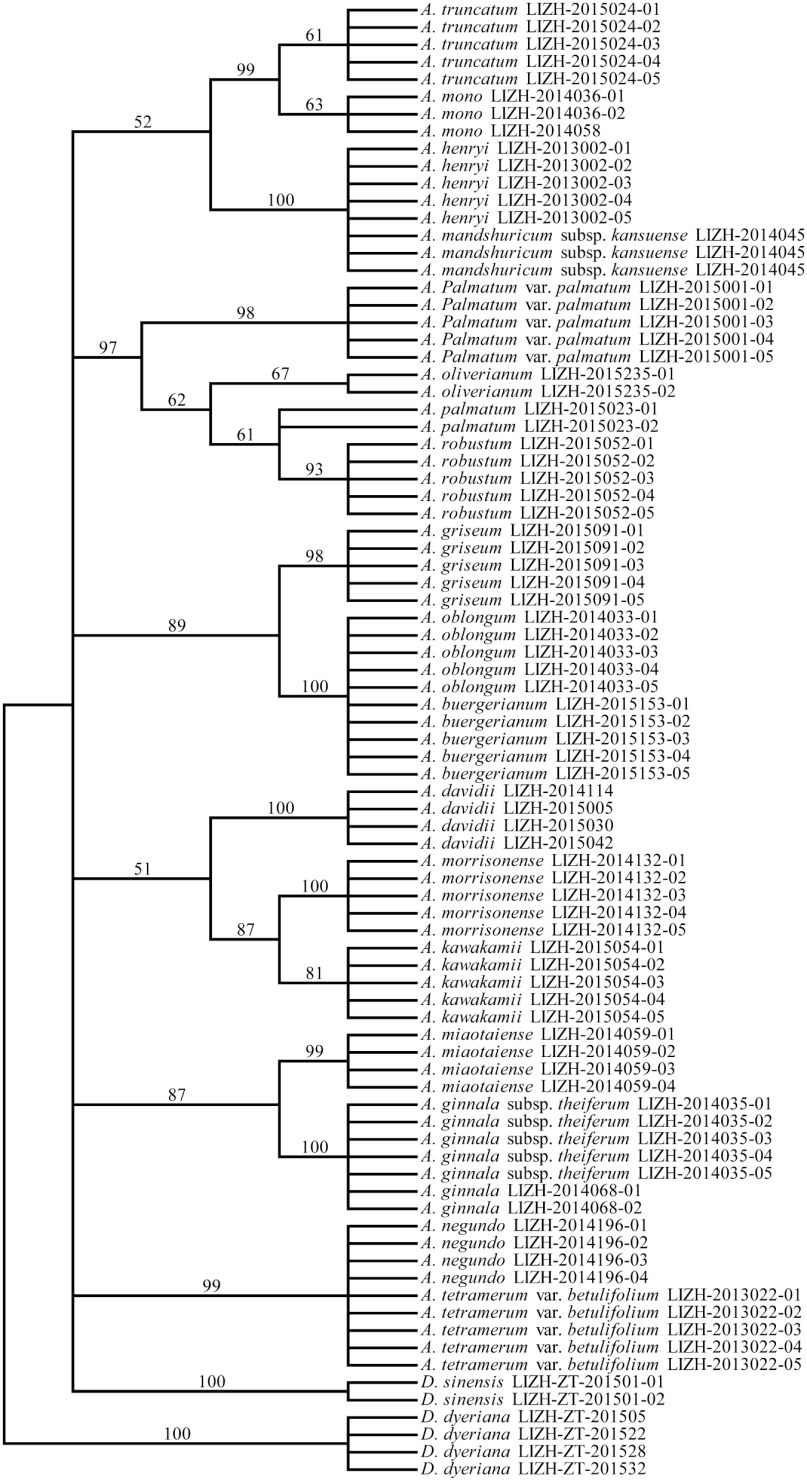
**Neighbor-Joining (NJ) tree of 21 Aceraceae species based on the combination of all three chloroplast (***rbc***L, ***mat***K, and ***trn***S-***trn***G) regions**.

**Figure 5:**
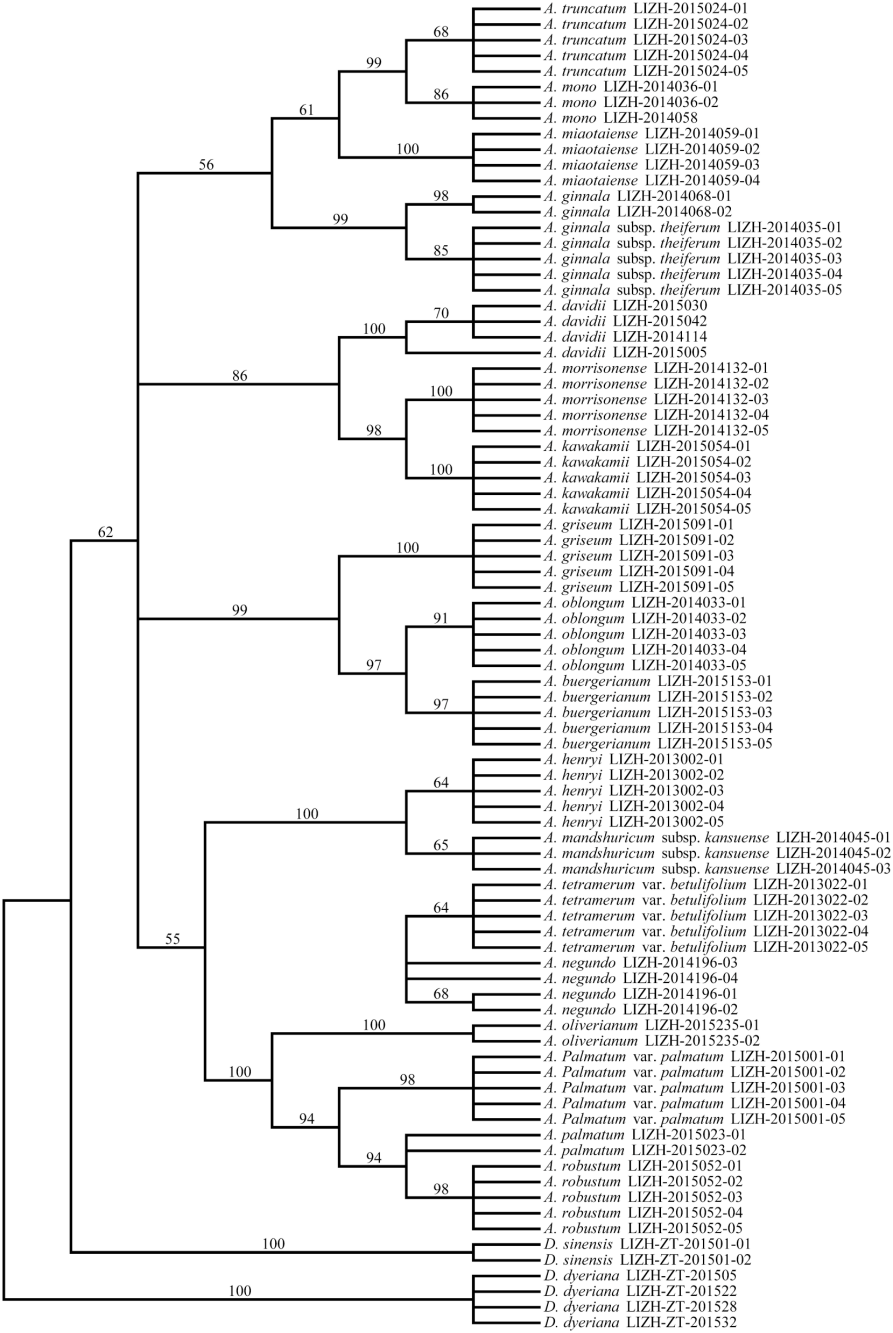
**Neighbor-Joining (NJ) tree of 21 Aceraceae species based on the combination of all single barcodes (***rbc***L, ***mat***K, ***trn***S-***trn***G, and ***ITS***)**.

To evaluate the species discrimination power of the four single barcoding markers and the eight combinations of two or more markers, two different analyses were used. In the Tree-Building and PWG-Distance analyses, there were consistent results in the species discrimination power for all markers (Figure 3). Generally, species forming separate clusters in the tree with bootstrap support >50% were considered to be discriminated. In the single chloroplast marker analysis, *mat*K had a relatively higher percentage of successful species discrimination (33.33%), followed by *trn*S-*trn*G (23.81%) and *rbc*L (8.11%). The percentage of species discrimination ranged from 23.81% (R+T) to 49.14% (M+T) for combinations of two chloroplast markers, and R+M provided successful species discrimination of 38.71%. Furthermore, the discrimination rate was 57.14% for combinations of three chloroplast markers (R+M+T) (Figure 3). The combinations of *ITS* plus one or three chloroplast barcodes significantly improved the species resolution (Figures 4, 5). The combinations T+I (90.5% resolution) and R+M+T+I (90.5% resolution) showed the highest percentage of successful species identification in the sampled Aceraceae species (Figure 3).

In addition, we evaluated the species discrimination power at the section level within Aceraceae (Figure S1). In the analysis of single barcode markers, the nuclear *ITS* fragment provided the highest resolution power (71.43%), followed by the three chloroplast DNA barcodes *mat*K (42.42%), *trn*S-*trn*G (37.5%) and *rbc*L (10.26%). Combinations of *ITS* plus one or three chloroplast DNA markers significantly improved species discrimination. The discrimination rate was 89.47% for the combination M+I. The combination R+M+T+I (90%) showed the highest percent identification at the section level within Aceraceae.

## Discussion

### Evaluation of barcode power in aceraceae

According to the CBOL Plant Working Group (2009), three main criteria should be satisfied in evaluating markers for plant DNA barcoding: (i) the presence of sufficient flanking sequences for developing appropriate universal barcoding primer markers, (ii) a relatively short nucleotide sequence to facilitate PCR amplification and sequencing, and (iii) significant genetic divergence at the species level. In our study, all samples of Aceraceae were successfully amplified and sequenced for the four DNA barcode candidates, with the exception of the slightly short sequences of *ITS*, which were manually edited. This result indicates a high universality for the DNA markers that we used here.

An ideal DNA barcode could provide significant species discrimination and identification (Feng et al., 2014; Gong et al., 2016; Nigro et al., 2016). We used two different analyses to evaluate the discrimination rates between species and sections within Aceraceae. The Tree-Building method is an analytical method used to generate a graphical representation of the results, which is useful for determining the power of a given combination of markers to discriminate between closely related species (Zhang et al., 2013). In addition, we used the PWG-Distance method to compute intra- and interspecific pairwise distances based on the Kimura two-parameter (K2P) model. Using these two approaches, we obtained similar results for species and section identification in Aceraceae.

### Low efficacy of the three chloroplast DNA markers

In the current study, the chloroplast barcoding markers or different combinations of the chloroplast barcoding markers (2-loci) had low identification power in the sampled Aceraceae species (Table 3). Among the single chloroplast markers, for two coding regions, *rbc*L had lower interspecific variation (0.0044) and species resolution (10.26%), but *mat*K had low interspecific variation (0.0086) and relatively high species resolution (42.42%). This result was similar to previous results, which showed very low divergence and low power to discriminate between species (Feng et al., 2013) for the *rbc*L region. These results imply that the two coding spacers, *rbc*L and *mat*K, are unsuitable for distinguishing the forest species in Aceraceae. The non-coding spacer *trn*S-*trn*G provided more interspecific distance (0.0268) than the two coding fragments (*rbc*L and *mat*K), but *trn*S-*trn*G provided lower species discrimination (33.3%). However, the debate regarding the use of non-coding regions in DNA barcoding is ongoing due to unreliable alignment of sequences with very high variation (Särkinen and George, 2013). In addition, some authors suggest that indel regions of non-coding DNA sequences provide important phylogenetic information and species discrimination power for some of the group, e.g., *Taxus* (Liu et al., 2011).

Recently, some studies have found that combinations of barcoding markers improved species delimitation power more than single candidate DNA barcodes (Feng et al., 2013; Zhang et al., 2014; Yan et al., 2015). The 2-locus combination of *mat*K+*rbc*L has been officially proposed as the core barcode for land plants (Clement and Donoghue, 2012; Wirta et al., 2016) based on the straightforward recovery of the *rbc*L region and the discriminatory power of the *mat*K region (CBOL Plant Working Group, 2009). In our study, this combination resulted in successful identification 38.71% of the time, which was higher than the combination of *rbc*L+*trn*S-*trn*G (23.81%) but lower than *mat*K+*trn*S-*trn*G (49.14%). From the above results, the core barcode region (*mat*K+*rbc*L) had higher species resolution than all single chloroplast barcoding markers. However, the identification power of the core barcode region is still lower than that of the *ITS* fragment. Our results indicate that the main barcode marker suggested by the CBOL Plant Working Group (2009) is not suitable for molecular identification of Aceraceae species and needs further investigation.

### The nuclear *ITS* region is a more promising barcode in aceraceae

In our study, the *ITS* marker showed the highest interspecific and intraspecific divergences (0.0474 and 0.0062, respectively) and the highest species resolution (50%) of all single DNA barcode candidates. This high resolution was also found in previous research (e.g., Li et al., 2011; Liu et al., 2016; Wirta et al., 2016), for example, in *Rhododendron* (Yan et al., 2015), Venus Slipper (Guo et al., 2016) and in Rosewood (Hartvig et al., 2015). These previous results showed that the *ITS* marker performed well as a single barcode for species identification, although the *ITS* marker was considered to have drawbacks, including incomplete lineage sorting and homogeneous concerted evolution (Liu et al., 2011, 2016; Wang et al., 2011; Wirta et al., 2016). However, in a recent study, Li et al. (2011) found that the nuclear *ITS* barcode had high species discrimination power, and they proposed that it should be incorporated into the core barcode marker for land plants.

In this study, the nuclear *ITS* fragment was shorter and had a higher evolutionary rate than the chloroplast DNA regions (*ITS*: 61.7, 137 mutation sites out of 346 bp; three chloroplast markers combined: 71.4%, 93 mutation sites out of 1499 bp; Table 3). Compared to the mode of uniparental inheritance of chloroplast DNA regions, the nuclear *ITS* region was a biparentally inherited marker, and its genetic information was transferred by pollen and seed. Thus, the *ITS* fragment has a larger effective population size than the chloroplast DNA regions, which makes the *ITS* marker is not easily quicker to complete lineage sorting than the cpDNA markers (Wachowiak et al., 2011). However, the nuclear *ITS* marker showed a higher evolutionary rate and higher inter-species differentiation than the chloroplast regions, and the nuclear *ITS* region in plants comprises multiple (reiterated) copies and usually undergoes concerted evolution (Alvarez and Wendel, 2003; Liu et al., 2016). During this process, different copies become homogenized to the same sequence type (becoming almost identical types) as a result of mechanisms such as high-frequency unequal crossing over or gene conversion (Alvarez and Wendel, 2003; Hartvig et al., 2015; Wirta et al., 2016). Therefore, this region may have accumulated more interspecific differentiation, comparable even to that experienced by speciation genes (Alvarez and Wendel, 2003; Guo et al., 2016; Wirta et al., 2016). Considering all characteristics of the nuclear *ITS* fragment, such as high mutation rate and rapidly concerted evolution, this nuclear gene fragment facilitates higher species discrimination power than chloroplast DNA regions in Aceraceae.

### A combined nuclear and chloroplast DNA barcode is an effective method of species delimitation of aceraceae

In the present study, we found that among sampled Aceraceae species, using a combination of the *ITS* fragment and a single chloroplast region usually significantly improved the discrimination efficiency (Figure 3), T+I (90.5% resolution) and R+M+T+I (90.5% resolution) differentiated the highest portion of species in Aceraceae (Figures 4, 5). This is in agreement with previous works on DNA barcoding of angiosperm taxa, e.g., *Alnus* (Betulaceae; Ren et al., 2010) and *Lamium* (Lamiaceae; Krawczyk et al., 2014). This result may have been caused by the uniparentally inherited chloroplast DNA fragment and the biparentally inherited nuclear *ITS* marker having different evolutionary modes and tracking different evolutionary histories. A combination of these markers improves species identification power and strengthens our understanding of evolutionary dynamics in plants (Saeki et al., 2011). However, in the present study, for the analysis of the discrimination rate of DNA barcoding combinations T+I and R+M+T+I, the number of sampled Aceraceae species was only 21, and the small number of collected individuals and species may have caused the high estimate of species resolution. In addition, we did not exclude the effect of biological characters in Aceraceae, e.g., complex sex systems and the dispersal of shorter distances among individuals within species, and these may have caused the high level of species identification (Liao et al., 2010; Zhang et al., 2010; Saeki et al., 2011). More samples and species should be collected for further assessment of phylogenetic relationships and the performance of DNA barcoding in the future.

### Implications for the phylogenetic relationships of aceraceae species

Phylogenetic identification and recognition of species are the keystones of biology (Moritz, 1994; Wirta et al., 2016). In the past, biologists identified phylogenetic relationships and positions of biological specimens mainly based on morphological features, such as leaves, flower shape, size, and color. However, traditional taxonomy has at least two drawbacks: first, if the specimens are damaged or lack sufficient diagnostic characters, the specialists may be unable to make accurate identifications (Yan et al., 2015); second, morphologically and taxonomically difficult groups sometimes require an experienced professional taxonomist to address species identification (Li et al., 2011; Liu J. et al., 2014). Such taxonomists need training and experience, which require much time, effort and money. With the development of molecular biology, the rise of DNA barcoding is expected to mitigate this dilemma, at least in part. DNA barcoding is one method that uses standard molecular techniques to achieve rapid and accurate species identification by a short DNA sequence (Hebert et al., 2003; Hebert and Gregory, 2005; Liu et al., 2013; Krawczyk et al., 2014). This method to remedy the limitations of a traditional morphology-based identification system will allow for more rapid progress in traditional taxonomic work (Kress et al., 2005; Liu J. et al., 2014; Nigro et al., 2016).

In the present study, we constructed the phylogenetic tree of the family Aceraceae by combining different DNA barcoding markers (Figures 4, 5). For the combination of all four DNA fragments (R+M+T+I), greater resolution in the NJ tree was achieved with higher bootstrap supports than with the combination of three chloroplast DNA markers (R+M+T). In the phylogenetic analyses, Aceraceae formed a monophyletic group with moderate bootstrap support. Many sections were resolved as monophyletic, with the exception of sect. *Trifoliata*. The monophyly formed by sect. *Palmata* and sect. *Microcarpa* was strongly supported with a high bootstrap value (100%). These two sections share many morphological characters, such as 4-paired bud scales, typically palmate leaves, and corymbose inflorescences. de Jong (1994) and Ogata (1967) combined them into one section, sect. *Palmata*. This result was confirmed by a previous study of nucleotide variations of chloroplast *trn*L-*trn*F and nuclear *ITS* regions (Tian et al., 2002). In addition, the close relationship between sect. *Trifolia* and sect. *Integrifolia* was supported with a high bootstrap value (99%) in the NJ tree. However, some species of the sections *Cissifolia, Negundo, Trifoliata* did not cluster together; *A. henryi* of section *Cissifolia* was closely related to *A. mandshuricum* subsp. *kansuense* of section *Trifoliata*, and the two species formed a clade with high bootstrap support, and *A. negundo* of section *Negundo* and *A. tetramerum Pax* var. *betulifolium* of section *Arguta* were clustered together (Figures 4, 5). We speculate that the hybridization and/or introgression among species from different sections may have caused the non-monophyletic clade within Aceraceae. Frequent inter-hybridization has been reported in Aceraceae (Liao et al., 2010; Zhang et al., 2010; Saeki et al., 2011). These characteristics might have caused the low resolution at the section level (Figure S1). We found the resolutions of the combinations M+I (89.47%) and R+M+T+I (90%) at the section level were lower than at the species level (M+ I: 90.5%, R+M+T+I: 90.5%).

In conclusion, we support the use of the *ITS* marker as a supplementary barcode in plants, while the performance of *ITS* should be evaluated in extensive trials in different plant groups (Liu et al., 2013; Liu J. et al., 2014; Wirta et al., 2016). The pragmatic solution to a complex trade-off between universality, sequence quality, discrimination, and cost, is the combination of T+I (90.5% species resolution), the most efficient and economical marker among single or combined DNA barcodes when identifying tree species in Aceraceae. More samples and species should be collected for further assessment of phylogenetic relationships and the performance of DNA barcoding.

## Author contributions

ZHL conceived and designed the research. YH, DD performed the experiments. YJ, ZHL, GZ, YH and ZL contributed materials/analysis tools. YH, ZHL wrote the paper. DD, XM and ZHL revised the paper. All authors read and approved the final manuscript.

### Conflict of interest statement

The authors declare that the research was conducted in the absence of any commercial or financial relationships that could be construed as a potential conflict of interest.

## Supplementary material

Figure S1 **Species discrimination rate of all tested single- and multi-locus barcodes at the section level in Aceraceae**. R, *rbc*L; M, *mat*K; T, *trn*S-*trn*G; I, *ITS*.

Table S1 **Samples for testing potential barcodes and accession numbers in GenBank**.

Supplementary Material Data Sheet **Alignments of DNA sequences of all tested 12 single- and multi-locus barcodes**. Sheet1, *ITS*; Sheet2, *mat*K+*ITS* (M+I); Sheet3, *mat*K+*trn*S-*trn*G (M+T); Sheet4, *mat*K; Sheet5, *rbc*L+ *ITS* (R+I); Sheet6, *rbc*L+ *mat*K (R+M); Sheet7, *rbc*L+*mat*K+*trn*S-*trn*G (R+M+T); Sheet8, *rbc*L+*mat*K+*trn*S-*trn*G+*ITS* (R+M+T+I); Sheet9, *rbc*L++*trn*S-*trn*G (R +T); Sheet10, *rbc*L; Sheet11, *trn*S-*trn*G+*ITS* (T+I); Sheet12, *trn*S-*trn*G.
