# Supplementary figures and images for: Efficient Identification of the Forest Tree Species in Aceraceae Using DNA Barcodes
Source: Front Plant Sci. 2016 Nov 16;7:1707. doi: 10.3389/fpls.2016.01707 (PMC5110567; doi:10.3389/fpls.2016.01707)

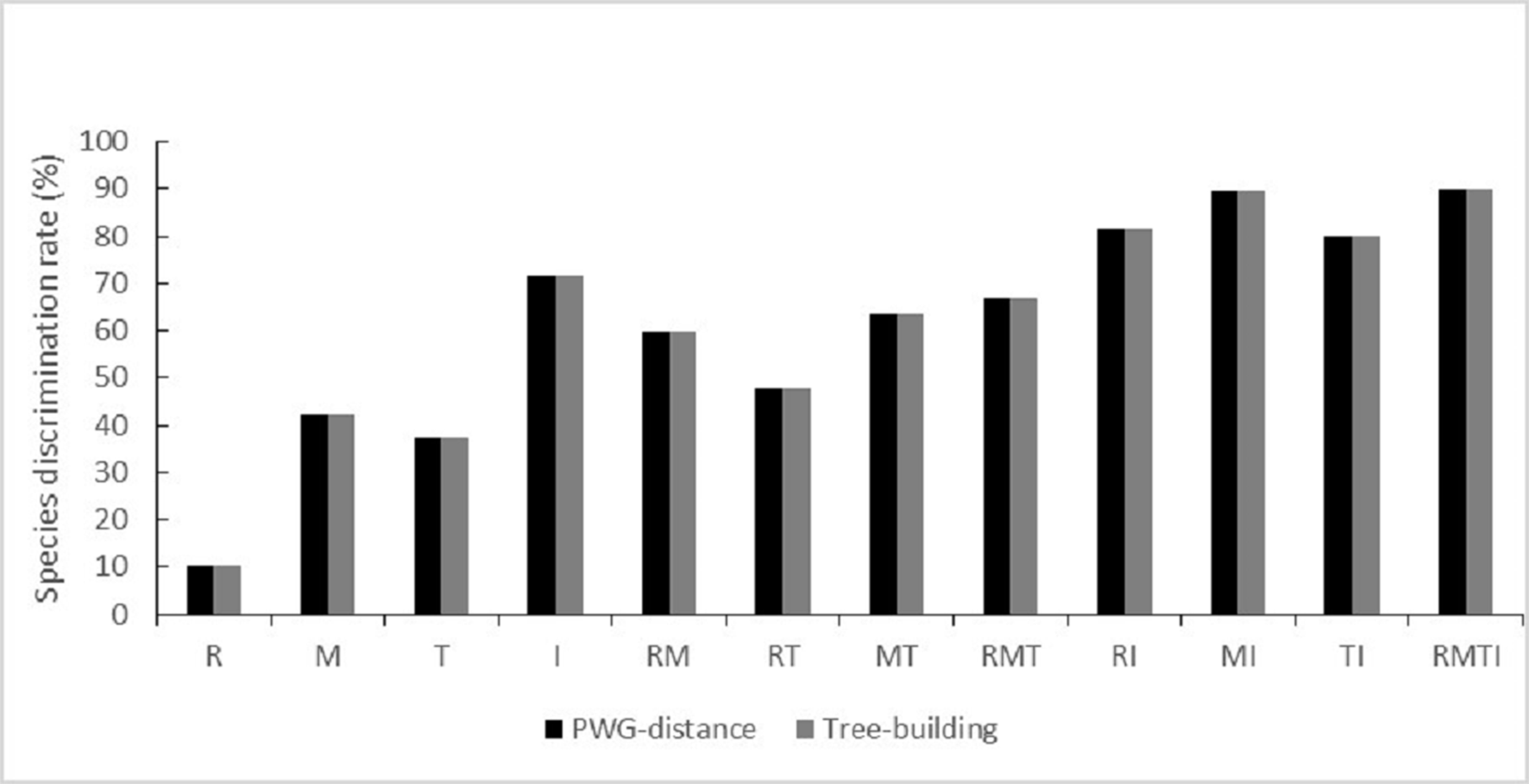

Supplement: Figure S1 — Species discrimination rate of all tested single- and multi-locus barcodes at the section level in Aceraceae. R, rbcL; M, matK; T, trnS-trnG; I, ITS. [file Image1.TIF]
