# Supplementary material for: Efficient Identification of the Forest Tree Species in Aceraceae Using DNA Barcodes
Source: Front Plant Sci. 2016 Nov 16;7:1707. doi: 10.3389/fpls.2016.01707 (PMC5110567; doi:10.3389/fpls.2016.01707)
Supplement: Table S1 — Samples for testing potential barcodes and accession numbers in GenBank. [file Table1.docx]

Table S1 Samples for testing potential barcodes and accession numbers in GenBank.

| Section | Species | *rbc*L |  |  |  | *mat*K |  |  |  | *ITS* |  |  |  | *trn*S*-trn*G |
| --- | --- | --- | --- | --- | --- | --- | --- | --- | --- | --- | --- | --- | --- | --- |
| section *Acer* L. | *A. heldreichii* Orph. ex Boiss. |  |  |  |  |  |  |  |  | AY605301.1 | AY605302.1 | AY605303.1 | AY605304.1 |  |
|  | *A. trautvetteri* Medw. | DQ978438.1 |  |  |  |  |  |  |  | AM238281.1 | AM238282.1 | AM238283.1 | AF401126.1 |  |
|  | *A. velutinum* Boiss. |  |  |  |  |  |  |  |  | AM238286.1 | AM238287.1 | AM238288.1 | AM238289.1 |  |
|  |  |  |  |  |  |  |  |  |  | AM238290.1 | AM238291.1 | AM238292.1 | AY605356.1 |  |
|  |  |  |  |  |  |  |  |  |  | AY605357.1 | AY605358.1 | AY605359.1 | AY605360.1 |  |
|  |  |  |  |  |  |  |  |  |  | AY605361.1 | AY605362.1 |  |  |  |
|  | *A. pseudoplatanus* L. | KJ204286.1 | DQ978425.1 | HM849739.1 | FN689357.1 | KU550002.1 | KU550003.1 | AJ438780.1 | AJ438781.1 | AM238254.1 | AM238256.1 | AM238257.1 | AM238259.1 |  |
|  |  |  |  |  |  | HM850604.1 | HE967335.1 | KJ204427.1 |  | AM238264.1 | AM238275.1 | AM238276.1 |  |  |
| section *Arguta* (Rehd.) E. Murray | *A. acuminatum* Wall. ex D. Don | DQ978393.1 |  |  |  |  |  |  |  | AY605370.1 | AY605371.1 | AY605372.1 |  |  |
|  | *A. argutum* Maxim. ex Miq | DQ978394.1 |  |  |  | AB872505.1 |  |  |  | AF401153.1 |  |  |  |  |
|  | *A. barbinerve* Maxim. ex Miq. | DQ978395.1 |  |  |  |  |  |  |  | AJ634569.1 |  |  |  |  |
|  | *A. tetramerum* Pax. var. *betulifolium* (Maxim.) Rehd. | KX264930 |  |  |  | KX264943 |  |  |  | KX264963 |  |  |  | KX264988 |
|  | *A. tetramerum* Pax |  |  |  |  |  |  |  |  | AF401154.1 |  |  |  |  |
| section *Carpinifolia* (Koidzumi) Momotani | *A. carpinifolium* Siebold et Zucc. |  |  |  |  |  |  |  |  | AF401148.1 | AY605379.1 | AY605380.1 | AY605381.1 |  |
| section *Cissifolia* Koidzumi | *A. cissifolium* (Siebold et Zucc.) K. Koch | DQ978404.1 |  |  |  | AB872509.1 |  |  |  | AF401140.1 | AF401141.1 | AF401142.1 |  |  |
|  | *A. henryi* Pax | KX264931 |  |  |  | KX264945 |  |  |  | AY605403.1 | AY605404.1 | AY605405.1 | AY605405.1 | KX264980 |
|  |  |  |  |  |  |  |  |  |  | KX264964 |  |  |  |  |
|  | *Dipteronia sinensis* Oliv | KX264925 |  |  |  | KX264944 |  |  |  | KX264962 |  |  |  | KX264989 |
|  | *D. dyeriana* Henry. | KX264934 |  |  |  | KX264951 | KX264952 |  |  | KX264974 |  |  |  | KX264991 |
| section *Distyla* E. Murray | *A. distylum* Siebold et Zucc | DQ978408.1 |  |  |  |  |  |  |  | AF401155.1 |  |  |  |  |
|  | *A. amamiense* T. Yamazaki |  |  |  |  | AB872503.1 |  |  |  |  |  |  |  |  |
| section *Ginnala* Nakai | *A. ginnala* Maxim. | KX264932 |  |  |  | KX264946 |  |  |  | KX264969 |  |  |  | KX264981 |
|  | *A. ginnala* Maxim. subsp. t*heiferum* (Fang) Fang. | KX264936 |  |  |  | KX264946 |  |  |  | KX264965 |  |  |  | KX264981 |
|  | *A. tataricum* L. | JF940633.1 | JF940634.1 | JF940635.1 |  | JF953000.1 | JF953001.1 | JF953002.1 | JF952999.1 | AF401146.1 |  |  |  |  |
| section *Glabra* Pax | *A. glabrum* Torr. | DQ978410.1 | KU043113.1 | KP903469.1 |  |  |  |  |  | AF401139.1 |  |  |  |  |
| section *Goniocarpa* Pojark. | *A. monspessulanum* L. | FN689377.1 |  |  |  | AJ438789.1 | AJ438790.1 | AJ438791.1 | AJ438792.1 | AF401127.1 |  |  |  |  |
|  | *A. opalus* Mill. | DQ978420.1 |  |  |  | KU549988.1 | AJ438787.1 |  |  | AF401128.1 |  |  |  |  |
| section *Hyptiocarpa* Fang | *A. laurinum* Hassk. | DQ978413.1 | KR528591.1 | KR528592.1 |  | KR530245.1 | KR530246.1 |  |  |  |  |  |  |  |
|  | *A. garrettii* Craib | KR528589.1 | KR528590.1 |  |  |  |  |  |  |  |  |  |  |  |
| section *Integrifolia* Pax | *A. buergerianum* Miq | KX264931 |  |  |  | KX264942 |  |  |  | AF401133.1 | AY605464.1 | AY605465.1 | AY605466.1 | KX264987 |
|  |  |  |  |  |  |  |  |  |  | KX264977 |  |  |  |  |
|  | *A. buergerianum* Miq. var. *formosanum* (Hayata ex Koidz.) Sasaki | DQ978396.1 |  |  |  |  |  |  |  | FN651690.1 | FN651691.1 | FN651692.1 | FN651693.1 |  |
|  | *A. cinnamomifolium* Hayata |  |  |  |  |  |  |  |  | DQ238467.1 | DQ238468.1 | DQ238469.1 | DQ238470.1 |  |
|  | *A. fabri* Hance | JF940624.1 | JF940625.1 | JF940627.1 | JF940626.1 | JF952990.1 | JF952991.1 | JF952992.1 | JF952993.1 |  |  |  |  |  |
|  |  | KP094586.1 | KP094587.1 | KJ440060.1 |  | KJ510960.1 | KP093653.1 | KP093654.1 |  |  |  |  |  |  |
|  | *A. laevigatum* Wall. | DQ978412.1 |  |  |  |  |  |  |  |  |  |  |  |  |
|  | *A. oblongum* Wall. ex DC. | DQ978419.1 | KX264928 |  |  | KX264942 |  |  |  | KX264959 |  |  |  | KX264987 |
|  | *A. paxii* Franch. |  |  |  |  |  |  |  |  | AF401132.1 |  |  |  |  |
|  | *A. poliophyllu*m W. P. Fang et Y. T. Wu |  |  |  |  |  |  |  |  | AF401134.1 |  |  |  |  |
| section *Lithocarpa* Pax | *A. kungshanense* W. P. Fang et C. Y. Chang |  |  |  |  |  |  |  |  | AF401143.1 |  |  |  |  |
|  | *A. pilosum* Maxim. | DQ978423.1 |  |  |  |  |  |  |  |  |  |  |  |  |
| section *Macrantha* Pax | *A. micranthum* Siebold et Zucc. |  |  |  |  | AB872517.1 |  |  |  |  |  |  |  |  |
|  | *A. tschonoskii* Maxim. | DQ978440.1 | AB872564.1 |  |  | AB872531.1 |  |  |  |  |  |  |  |  |
|  | *A. wardii* W. W. Smith |  |  |  |  |  |  |  |  | AF401159.1 |  |  |  |  |
|  | *A. tegmentosum* Maxim. | DQ978437.1 |  |  |  |  |  |  |  | AF401145.1 |  |  |  |  |
|  | *A. pectinatum* Wall. ex G. Nichols. | JF940630.1 | JF940631.1 | JF940632.1 |  | JF952996.1 | JF952997.1 | JF952998.1 |  |  |  |  |  |  |
|  | *A. pensylvanicum* L. | KP643912.1 | KP643776.1 | KJ841074.1 |  | KP210354.1 | KP642893.1 | KP643010.1 |  | AY605398.1 |  |  |  |  |
|  | *A. crataegifolium* Siebold et Zucc. | DQ978405.1 |  |  |  | AB872510.1 |  |  |  | AY605391.1 |  |  |  |  |
|  | *A. davidii* Franch. | JF940614.1 | JF940615.1 | JF940616.1 | JF940617.1 | JF952981.1 | JF952982.1 | JF952983.1 | JF952984.1 | AF401144.1 | KX264966 | KX264970 |  | KX264990 |
|  |  | JF940618.1 | JF940619.1 | JF940620.1 | JF940621.1 | JF952985.1 | JF952986.1 | JF952987.1 | JF952988.1 |  |  |  |  |  |
|  |  | JF940622.1 | KX264931 |  |  | KX264948 |  |  |  |  |  |  |  |  |
|  | *A. rufinerve* Siebold et. Zucc. | DQ978429.1 |  |  |  | AB872527.1 |  |  |  | AY605399.1 | AY605400.1 |  |  |  |
|  | *A. kawakamii* Koidzumi. | KX264930 |  |  |  | KX264949 |  |  |  | KX264971 |  |  |  | KX264983 |
|  | *A. morrisonense* Hayata | KX264925 |  |  |  | KX264947 |  |  |  | KX264968 |  |  |  | KX264982 |
| section *Macrophylla* Momotani | *A. macrophyllum* Pursh | DQ978414.1 |  |  |  |  |  |  |  | AF401156.1 | AY605385.1 | AY605386.1 | AY605387.1 |  |
|  |  |  |  |  |  |  |  |  |  | AY605388.1 |  |  |  |  |
| section *Microcarpa* Pojark. | *A. elegantulum* W. P. Fang et P. L. Chiu | HQ427191.1 |  |  |  |  |  |  |  |  |  |  |  |  |
|  | *A. olivaceum* W. P. Fang et P. L. Chiu |  |  |  |  | HQ427338.1 |  |  |  |  |  |  |  |  |
|  | *A. erianthum* Schwer | DQ978409.1 |  |  |  |  |  |  |  | AY605414.1 | AY605415.1 | AY605416.1 |  |  |
|  | *A. miaoshanicu*m Fang |  |  |  |  |  |  |  |  | AF401124.1 |  |  |  |  |
|  | *A. oliverianum* Pax. | KX264931 |  |  |  | KX264950 |  |  |  | KX264973 |  |  |  | KX264984 |
|  | *A. pubinerve* Rehd | KP094762.1 |  |  |  | KP093823.1 |  |  |  | AF401125.1\| |  |  |  |  |
|  | *A. tutcheri* Duthie | KP094923.1 |  |  |  | KP093979.1 |  |  |  |  |  |  |  |  |
|  | *A. ukurunduense* Trautv. et Mey. | DQ978402.1 | AB872565.1 |  |  | AB872532.1 |  |  |  |  |  |  |  |  |
| section *Negundo* (Boehmer) Maxim | *A. negundo* L. | DQ978417.1 | HQ589931.1 | EU676886.1 | EU676887.1 | EU749281.1 | HQ593152.1 | HM850603.1 | HE967334.1 | AF401142.1 | AY605406.1 | AY605407.1 | AY605408.1 | KX264988 |
|  |  | HM849738.1 | KT695524.1 | FN689355.1 | KX264929 | KX264943 |  |  |  | AY605409.1 | KX264960 | KX264961 |  |  |
| section *Palmata* Pax | *A. circinatum* Pursh | DQ978403.1 |  |  |  |  |  |  |  | AY605412.1 | AY605413.1 |  |  |  |
|  | *A. japonicum* Thunb. |  |  |  |  | AB872515.1 |  |  |  | AY605421.1 |  |  |  |  |
|  | *A. palmatum* Thunb. | DQ978421.1 | KX264925 |  |  | AB038174.1 | KP088937.1 | KX264938 |  | AF401123.1 | AB683972.1 | AB683975.1 | KX264955 | KX264978 |
|  | *A. Palmatum* Thunb. var. *palmatum* | KX264926 |  |  |  | KX264939 |  |  |  | KX264956 |  |  |  | KX264979 |
|  | *A. pseudosieboldianum* (Pax) Komarov | DQ978426.1 |  |  |  | KP088938.1 |  |  |  | AB683974.1 |  |  |  |  |
|  | *A. robustum* Pax | KX264925 |  |  |  | KX264940 |  |  |  | KX264957 |  |  |  | KX264978 |
|  | *A. shirasawanum* Koidz. |  |  |  |  | AB872528.1 |  |  |  | AY605427.1 | AY605428.1 |  |  |  |
|  | *A. sieboldianum* Miq. | DQ978433.1 | AB872562.1 |  |  | AB872529.1 |  |  |  |  |  |  |  |  |
|  | *A. tenuifolium* Koidz. | AB872563.1 |  |  |  | AB872530.1 |  |  |  |  |  |  |  |  |
| section *Parviflora* Koidzumi | *A. nipponicum* H. Hara | DQ978418.1 |  |  |  |  |  |  |  | AF401157.1 |  |  |  |  |
| section *Pentanphylla* Hu et Cheng | *A. pentaphyllum* Diels | DQ978422.1 |  |  |  |  |  |  |  |  |  |  |  |  |
| section *Platanoidea* Pax | *A. campestre* L. | KM360609.1 | JN892112.1 | JN892105.1 | JN890783.1 | KU549936.1 | JN894032.1 | JN894999.1 | KC584926.1 | AF401158.1 | AY605437.1 | AY605438.1 | AY605439.1 |  |
|  |  | FN689361.1 |  |  |  | AJ438793.1 | AJ438794.1 | AJ438795.1 | AJ438796.1 | AY605440.1 | AY605441.1 | AY605442.1 | AY605444.1 |  |
|  | *A. cappadocicum* Gled. |  |  |  |  |  |  |  |  | AF401138.1 | AY605445.1 |  |  |  |
|  | *A. miaotaiense* Tsoong. | KX264933 |  |  |  | KX264941 |  |  |  | KX264967 |  |  |  | KX264981 |
|  | *A. miyabei* Maxim. |  |  |  |  | AB872518.1 |  |  |  | AY605451.1 | AY605452.1 |  |  |  |
|  | *A. mono* Maxim. | KX264927 |  |  |  | KX264941 |  |  |  | AF241491.1 | AY605446.1 | AY605447.1 | AY605449.1 | KX264986 |
|  |  |  |  |  |  |  |  |  |  | AY605450.1 | JF980310.1 | U57772.1 | U57775.1 |  |
|  |  |  |  |  |  |  |  |  |  | KX264958 |  |  |  |  |
|  | *A. truncatum* Bunge. | KX264937 |  |  |  | KX264953 |  |  |  | KX264975 |  |  |  | KX264986 |
|  | *A. lobelii* Ten. | FN689360.1 |  |  |  |  |  |  |  |  |  |  |  |  |
|  | *A. platanoides* L. | DQ978424.1 | FN689356.1 | EU676888.1 |  | KU549936.1 |  |  |  | AF401136.1 | AY605453.1 | AY605454.1 | AY605455.1 |  |
|  |  |  |  |  |  |  |  |  |  | AY605456.1 | AY605457.1 |  |  |  |
| section *Trifoliata* Pax | *A. griseum* (Franch.) Pax. | KX264935 |  |  |  | KX264954 |  |  |  | KX264976 |  |  |  | KX264985 |
|  | *A. mandshuricum* Maxim. subsp. Kansuense (Fang et C. Y. Chang) Fang. | KX264931 |  |  |  | KX264945 |  |  |  | KX264972 |  |  |  | KX264980 |
|  | *A. triflorum* Komarov |  |  |  |  |  |  |  |  | AF401130.1 | AY605472.1 |  |  |  |
|  | *A. maximowiczianum* Miq. | DQ978415.1 |  |  |  | AB872516.1 |  |  |  | AJ698721.1 | AJ698722.1 |  |  |  |
| section *Rubra* Pax | *A. pycnanthum* K. Koch | DQ978427.1 |  |  |  | AB872526.1 |  |  |  |  |  |  |  |  |
|  | *A. rubrum* Pax | DQ978428.1 | DQ978429.1 | HQ589932.1 | KJ593171.1 | EU749284.1 | EU749285.1 | EU749286.1 | KJ592831.1 | AF401150.1 | AY605460.1 | AJ634580.1 | AJ634585.1 |  |
|  |  | KJ593172.1 | KJ593173.1 | KF977466.1 | EU676890.1 | KJ840846.1 |  |  |  |  |  |  |  |  |
|  |  | EU676891.1 | EU676892.1 | KJ841075.1 |  |  |  |  |  |  |  |  |  |  |
|  | *A. spicatum* Lam. | HQ589933.1 | DQ978434.1 |  |  | HQ593153.1 | KJ592832.1 |  |  | AF401122.1 | AY605431.1 | AJ634575.1 | AJ634576.1 |  |
| section *Saccarodendron* E. Murray | *A. saccharinum* L. | EU676893.1 | EU676894.1 | EU676895.1 | EU676896.1 | EU749289.1 |  |  |  | AF401151.1 | AY605462.1 | AY605463.1 |  |  |
|  | *A. saccharum* Marshall | KJ593174.1 | KJ593175.1 | KJ593176.1 | EU676898.1 | AY724265.1 | EU749291.1 |  |  | AF401152.1 |  |  |  |  |
|  |  | EU676899.1 | KJ841076.1 | EU676897.1 |  |  |  |  |  |  |  |  |  |  |
|  | *A. nigrum* Marsh | AY605322.1 | AY605323.1 | AY605324.1 |  |  |  |  |  |  |  |  |  |  |
|  | *A. grandidentatum* Nutt. |  |  |  |  |  |  |  |  | AM238323.1 | AM238324.1 | AY605297.1 | AY605298.1 |  |
|  |  |  |  |  |  |  |  |  |  | AY605299.1 | AY605300.1 |  |  |  |

Note：

*rbc*L (R): 66 species, 193 sequences.

*mat*K (M): 54 species, 166 sequences.

*trn*S-*trn*G (T): 21 species, 85 sequences.

*ITS* (I): 68 species, 231 sequences.

R+M: 47 species, 134 sequences.

M+T: 21 species, 85 sequences.

R+T: 21 species, 85 sequences.

R+M+T: 21 species, 85 sequences.

R+I: 46 species, 121 sequences.

M+I: 41 species, 119 sequences.

T+I: 21 species, 85 sequences.

R+M+T+I: 21 species, 85 sequences.
